# Proteomic Analysis of Cardiac Adaptation to Exercise by High Resolution Mass Spectrometry

**DOI:** 10.3389/fmolb.2021.723858

**Published:** 2021-09-01

**Authors:** Afnan Saleh Al-Menhali, Cali Anderson, Alexander V. Gourine, Andrey Y. Abramov, Alicia D’Souza, Morana Jaganjac

**Affiliations:** ^1^ Division of Medicine, University College London, London, United Kingdom; ^2^ Qatar Analytics and BioResearch Lab, Anti Doping Lab Qatar, Doha, Qatar; ^3^ Division of Cardiovascular Sciences, University of Manchester, Manchester, United Kingdom; ^4^ Centre for Cardiovascular and Metabolic Neuroscience, Department of Neuroscience, Physiology and Pharmacology, University College London, London, United Kingdom; ^5^ Department of Clinical and Movement Neuroscience, UCL Institute of Neurology, London, United Kingdom; ^6^ Division of Molecular Medicine, Rudjer Boskovic Institute, Zagreb, Croatia

**Keywords:** exercise, left ventricle, proteomics, oxidative stress, 4-hydroxynonenal

## Abstract

Regular exercise has many health benefits, among which is a significant reduction of cardiovascular risk. Although many beneficial effects of exercise are well described, the exact mechanisms by which exercise confers cardiovascular benefits are yet to be fully understood. In the current study, we have used high resolution mass spectrometry to determine the proteomic responses of the heart to exercise training in mice. The impact of exercise-induced oxidative stress on modifications of cardiomyocyte proteins with lipid peroxidation biomarker 4-hydroxynonenal (4-HNE) was examined as well. Fourteen male mice were randomized into the control (sedentary) group and the exercise group that was subjected to a swim exercise training program for 5 days a week for 5 months. Proteins were isolated from the left ventricular tissue, fractionated and digested for shotgun proteomics. Peptides were separated by nanoliquid chromatography and analyzed on an Orbitrap Fusion mass spectrometer using high-energy collision–induced dissociation and electron transfer dissociation fragmentation. We identified distinct ventricular protein signatures established in response to exercise training. Comparative proteomics identified 23 proteins that were upregulated and 37 proteins that were downregulated with exercise, in addition to 65 proteins that were identified only in ventricular tissue samples of exercised mice. Most of the proteins specific to exercised mice are involved in respiratory electron transport and/or implicated in glutathione conjugation. Additionally, 10 proteins were found to be modified with 4-HNE. This study provides new data on the effects of exercise on the cardiac proteome and contributes to our understanding of the molecular mechanisms underlying the beneficial effects of exercise on the heart.

## Introduction

In the early 1950s, Morris and others showed the association between physical activity and reduced deaths from coronary heart disease, which led to increased scientific interest in the potential of physical activity to combat diseases (Morris et al., 1953). Current knowledge shows a clear link between an active lifestyle and overall health. Physical exercise is found to benefit the heart as it increases aerobic fitness (VO_2max_), enhances contraction, and accelerates relaxation, as well as decreasing the risk of developing cardiomyopathies (Roof et al., 2013). It is found that different types, intensities, durations, and frequencies of exercise interventions are crucial for cardiovascular health (Gevaert et al., 2020). Endurance exercise can trigger several adaptational mechanisms resulting in exercise-induced cardioprotection changes. Those changes include an enhanced antioxidant defense system with increased expression of glutathione peroxidase-1 and manganese superoxide dismutase. Moreover, cardiac function is improved due to changes in the expression of enzymes involved in energy metabolism (Burniston and Hoffman, 2011).

Many studies suggest the beneficial effects of exercise-induced oxidative stress. It is suggested that the released reactive oxygen species (ROS) post–physical exercise can lead to cardiac adaptation, including protection against infarction (Frasier et al., 2011). At a molecular level, excess oxidants can damage macromolecules. Lipids are one of the target sites of ROS-induced damage, where it was found that maximal and supra-maximal exercise can significantly increase lipid peroxidation (Mohamed et al., 2016).

Lipid peroxidation can result in the formation of many reactive species as secondary products, known as reactive carbonyl compounds (RCCs), due to the highly reactive carbonyl group. Those secondary products have high stability with an average half-life of minutes or even hours, compared to the short half-life of ROS, which can only last for nanoseconds or milliseconds (Jaganjac et al., 2016). As a result, RCCs can diffuse through cellular membranes and attack biomolecules that are far away from the site of origin (Pamplona, 2011; Jaganjac et al., 2013). Among all secondary products studied, of particular biochemical and biomedical relevance is 4-hydroxynonenal (4-HNE) (Živković et al., 2005).

The physiological levels of 4-HNE vary between different cell types or tissues. Physiological concentrations of 4-HNE are in the submicromolar range, and for the heart samples, a 4-HNE concentration of around 0.25 nmol/mg of tissue (Nakagawa et al., 2014) or 6 nmol/mg of protein (Beneš et al., 2013) was reported. 4-HNE can bind to the nucleophilic amino acid side chain of proteins *via* the Michael addition or the Schiff base formation modifying protein function (Zarkovic et al., 2013). It is crucial for cells to control 4-HNE concentrations, as low concentrations are needed for normal physiological processes, while high concentrations can have detrimental effects (Jaganjac et al., 2020a).

In this study, conducted in experimental animals (mice), we aimed to determine the effect of intense exercise training on the left ventricle proteome and on the formation of 4-HNE protein adducts by using in-depth proteomics.

## Materials and Methods

### Animals

Experiments were performed on 14 male C57BL/6J mice that were 8–10 weeks old, with water and food given *ad libitum*. All experiments were performed in accordance with the European Commission Directive 2010/63 (European Convention for the Protection of Vertebrate Animals Used for Experimental and Other Scientific Purposes) and the United Kingdom Home Office (Scientific Procedures) Act (1986) with project approval by the Institutional Animal Welfare and Ethical Review Committee of the University of Manchester (PBA6A87CA).

### Training Protocol and Sample Collection

Animals were randomized into two groups: a sedentary group and an exercised group that was trained by swimming for 5 months, for 60 min per day for 5 days per week. After 5 months, the animals were terminated by cervical dislocation, following which the heart was rapidly excised. A 2-mm biopsy of the left ventricle free wall was sampled, approximately midway between the base and the apex, and stored at −80°C until further analysis. At the time of sample collection, the mice were 7–7.5 months old.

### Protein Isolation and Digestion

Left ventricular tissue was homogenized in a glass homogenizer in ice-cold lysis buffer (20 mM HEPES, 20 mM NaCl, 5 mM EDTA, 1% w/v CHAPS, and protease inhibitors). 7 ml of buffer was added per 1 g of tissue. Lysates were then sonicated for 10 min, followed by centrifugation at 14,000 × *g* for 10 min at +4°C. Supernatants were collected into new Eppendorf tubes, and protein concentration was determined using a BCA Protein Assay Kit (Pierce).

Protein samples were prepared for proteomics analysis in a similar manner to that described before (Al-Thani et al., 2018). Briefly, normalized protein samples were electophoretically separated on an SDS-PAGE using non-reducing sample buffer and whole lines were excised and divided into 8 equal parts that were then reduced with 10 mM dithiothreitol and alkylated with 100 mM iodoacetamide, followed by overnight digestion at 37°C with 20 ng/μl Trypsin Gold MS grade (Promega). The mixture of 45% water, 50% acetonitrile, and 5% formic acid was used to extract peptides.

### Shotgun Proteomics

Samples were analyzed using an Orbitrap Fusion Tribrid mass spectrometer (Thermo Scientific, Waltham, United States) coupled with an Easy n-LC II (Thermo Scientific, Waltham, United States) for nano-LC gradient separation. Thermo Xcalibur (version 3.0) software was used to control instrument setup. The nanoelectrospray ionization (NSI) mode was used, which is the preferred ionization mode for peptides and proteins. Peptide mixtures were separated on a reverse-phase C18 column (25 cm, 75 μm, 2 μm, Acclaim RSLC C18, Thermo Scientific, Waltham, United States) attached to a pre-trapping C18 column (2 cm, 75 μm, 3 μm, Acclaim, Thermo Scientific, Waltham, United States), and elution was carried out at a constant flow rate of 300 nl/min over a 108-min step gradient. Solvents used for separation were solvent A (HPLC-grade water with 0.1% (v/v) formic acid) and solvent B (HPLC-grade acetonitrile with 0.1% (v/v) formic acid). The separation gradient was set to 5% B for 5 min, 5–37% B for 90 min, 37–80% B for 4 min, 80% B for 2 min, 80–5% B for 2 min, and 5% B for 5 min.

The Orbitrap Fusion Tribrid analysis was set to data-dependent acquisition (DDA), using an Orbitrap mass analyzer to acquire the full MS spectra and an IonTrap to acquire the MS/MS fragment ion spectra. First full-scan mode acquisition was performed with a resolution of 120,000 orbitrap and a scan range of 400–1,600 m/z. Automatic gain control (AGC) was set at 200,000 with an injection time of 100 ms. This scan mode was carried out by enabling a monoisotopic precursor selection (MIPS) filter and dynamic exclusion with a duration of 30 s and a mass tolerance of 10 ppm. This is followed by two different second scan modes based on decisions of two scan event types. Scan event type 1 uses higher-energy collision–induced dissociation (HCD) fragmentation at the quadrupole isolation mode, which generates b and y ions, with an isolation width of 2 m/z and a collision energy of 30% and at 10,000 AGC and a maximum injection time of 70 ms. The neutral loss ion is triggered with masses of 52 or 78 m/z from the 156-Da 4-HNE–modified peptide by the Michael addition or masses of 46 or 69 m/z from the 138-Da 4-HNE–modified peptide by the Schiff bases (Carini et al., 2004). Selected peptides are further fragmented by electron transfer dissociation (ETD), which generates c and z ions and cleaves the amide group, leaving the side chain intact, using the quadrupole isolation mode with an isolation width of 2 m/z and a collision energy of 30% and at 10,000 AGC and a maximum injection time of 70 ms. Meanwhile, scan event type 2 is HCD fragmentation at the quadrupole isolation mode with an isolation width of 1.6 m/z and a collision energy of 30% and at 10,000 AGC and a maximum injection time of 70 ms.

### Data Processing and Analysis

The raw data generated by the Orbitrap Fusion were processed using Proteome Discoverer 2.2 (Thermo Scientific, San Jose, California, United States). The spectrum selector filter was assigned with 350 Da as the minimum precursor mass and 5,000 Da as the maximum precursor mass. The MS/MS spectra search was carried out using SEQUEST HT search algorithms against the UniProt *Mus musculus* (Mouse) protein database, and the FASTA file was retrieved on the 9^th^ of October, 2017. Two scan event filters were used: one with the HCD activation type, followed by Sequest HT search with the following parameters: full trypsin digestion, a maximum of 2 missed cleavages sites, a minimum peptide length of 6 residues, a tolerance of 20 ppm for precursor mass and 0.6 Da for fragment mass, dynamic modification of oxidation of methionine (+15.995 Da), and static modification of carbamidomethyl of cysteine (+57.021 Da). The second scan event filter included the ETD activation type with 1,000 set as the maximum collision energy, followed by another Sequest HT search using a comprehensive workflow that included additional dynamic modifications: 4-HNE adduction to cysteine, histidine, lysine, leucine, methionine, and arginine (+156.115 Da); 4-HNE + H_2_ for the Michael adducts in cysteine, histidine, lysine, leucine, methionine, and arginine (+158.131 Da); and 4-HNE-H_2_O for the Schiff bases in cysteine, histidine, lysine, leucine, methionine, and arginine (+138.104 Da). The target decoy PSM validator node was used for peptide validation with an FDR of 0.01. For precursor ion quantification, label-free quantification using the Minora Feature Detection was used, followed by statistical analysis using *t*-test (*p* < 0.05) to measure proteins that were significantly upregulated in the exercised group compared to the control group. On the other hand, the consensus workflow parameters used were as follows: a peptide validator with an automatic validation mode of 0.01 FDR, peptides and proteins were searched using a high-confidence peptide filter, and the precursor ion quantifier calculated abundance based on the intensity. For high confidence, XCorr values were set to at least 1.8 for singly, 2.4 for doubly, 2.5 for triply, and 2.6 for quadruply or more charged peptides. The mass spectrometry proteomics data have been deposited in the ProteomeXchange Consortium *via* the PRIDE (Perez-Riverol et al., 2019) partner repository with the dataset identifier PXD026558.

Only cardiac proteome changes detected in six or more samples per group were considered for further data visualization and interpretation. Differential expression analysis was performed using Perseus 1.6.15.0. (Tyanova et al., 2016) according to the protocol of Tyanova & Cox (Tyanova et al., 2018), and the FDR value was set to 0.05. The results are reported with the corrected *p*-value (q-value).

Data analysis of proteins identified in both groups was performed using InfernoRDN v1.1.7234 software (Polpitiya et al., 2008). Data were log-transformed and normalized by central tendency adjustment, followed by partial least square (PLS) analysis. Moreover, variables upregulated or downregulated with exercise with *p*-values ≤ 0.05 were visualized on the heat map, while all proteins that met the criteria and were identified in the database are plotted in a volcano plot.

Protein ANalysis THrough Evolutionary Relationships (PANTHER) classification system software (version 14.0) was used to analyze changes in different classes of proteins based on identified proteins and to analyze gene ontology annotations available in the UniProt database (Mi et al., 2019). The Reactome 3.7 database of reactions, pathways, and biological processes with database release 73 was used for pathway analysis of left ventricular proteins upregulated or only identified in the exercised group (Haw and Stein, 2012).

## Results

High resolution mass spectrometry was used to study changes in the left ventricular tissue proteome after long-term intensive swimming exercise. The total number of identified proteins in the control and the exercise group was 1,839 and 1,915, respectively, while the total number of identified peptides was 10,092 and 10,129, respectively. Further analyses were based on the criteria that the protein/peptide must be common for ≥6 tissue samples of the same group of animals. Based on that criteria, a total of 804 proteins were identified in the control group with a total number of 2,913 peptides, while 816 proteins were found in the exercised group with a total of 3,254 peptides.

**FIGURE 1:**
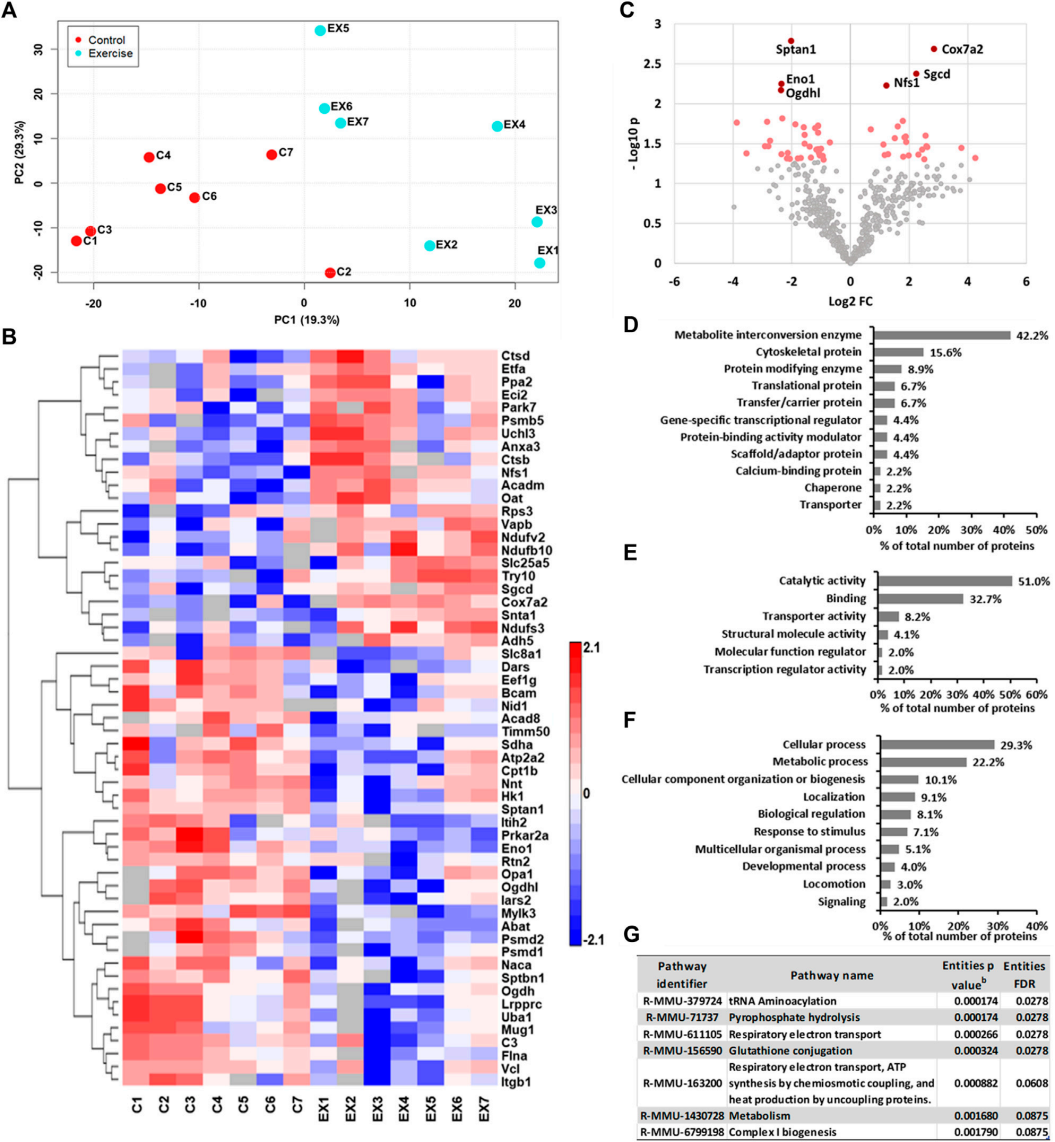
**(A)** PLS scatter plot showing the difference between control and exercised mice. **(B)** Heat map of semiquantitative assessment of ventricular proteins upregulated/downregulated with exercise. **(C)** Volcano plot of differentially expressed left ventricular tissue proteins between the control and exercised groups. The significantly upregulated and downregulated proteins are indicated with red dots, while those with *p* < 0.01 are indicated with dark red dots. **(D–F)** Repartition of upregulated proteins identified in the left ventricle of the heart tissues of exercised mice by **(D)** protein classes, **(E)** molecular functions, and **(F)** biological processes using the bioinformatics algorithms of the PANTHER classification system (version 15.0). **(G)** Biological pathways regulated by proteins altered by exercise. Proteins upregulated in ≥6 samples were analyzed using the Reactome database for *Mus musculus*, and only those with Entities FDR <0.1 are shown. ^b^p value: represents the probability that the overlap between the query has occurred by1 chance.

To evaluate the impact of exercise on the cardiac proteome, we have used the comparative proteomics and label-free proteomics approach. A total of 125 proteins displayed statistically significant changes in the ventricular tissue of the swimming exercise group compared to the control group (Tables 1, 2). Table 1 lists 65 proteins that were identified in ≥6 ventricular tissue samples of exercised mice, while the same were not identified in the control mice. Proteins that were identified in 6/7 samples for each group were selected for PCA analysis, to determine the multivariate exercise signature of proteins (Figure 1A). The semiquantitative assessment of proteins upregulated/downregulated with exercise identified 23 proteins that were upregulated and 37 that were downregulated with exercise (Figure 1B), and details of each are included in Table 2. The changes in the protein abundances are shown using a volcano plot in Figure 1C. The largest increase following exercise training was observed for mitochondrial cytochrome c oxidase subunit 7A2, cathepsin B, and ubiquitin carboxyl-terminal hydrolase isozyme L3. In addition, the most significant exercise-induced upregulation was observed for mitochondrial cytochrome c oxidase subunit 7A2, delta-sarcoglycan, and mitochondrial cysteine desulfurase. PLS analysis demonstrated good separation between the two groups with distinct protein signatures in the ventricular samples of exercised animals.

**TABLE 1:** Left ventricular tissue proteins identified only in samples of exercised mice. Proteins identified in ≥6 left ventricular tissue samples isolated from exercised animals but in none of the samples of the control group are listed. The table shows the UniProt name of the protein, main description, gene coding of the protein, number of amino acids (AAs), and molecular weight (MW).

UniProtKB accession	Gene	Description	# AAs	MW [kDa]	Number of positive samples
G3UYJ7	Gm20441	Predicted gene 20441 (fragment)	252	28.6	7
Q9WUK2	Eif4h	Eukaryotic translation initiation factor 4H	248	27.3	7
Q99JR6	Nmnat3	Nicotinamide/nicotinic acid mononucleotide adenylyltransferase 3	245	27.7	7
Q9D7J4	Cox20	Cytochrome c oxidase protein 20 homolog	117	13.2	7
Q9D819	Ppa1	Inorganic pyrophosphatas	289	32.6	7
Q9R1P3	Psmb2	Proteasome subunit beta type-	201	22.9	7
O88587	Comt	Catechol O-methyltransferase	265	29.5	7
E9QMD2	Ube2ql1	Ubiquitin-conjugating enzyme E2Q-like protein 1	304	32.6	7
Q6ZWQ0	Syne2	Nesprin-2	6,874	782.2	7
Q99JI6	Rap1b	Ras-related protein Rap-1b	184	20.8	7
P68369	Tuba1a	Tubulin alpha-1A chain	451	50.1	7
Q8K2C6	Sirt5	NAD-dependent protein deacylase sirtuin-5, mitochondrial	310	34.1	7
O09131	Gsto1	Glutathione S-transferase omega-1	240	27.5	7
P10649	Gstm1	Glutathione S-transferase Mu 1	218	26	7
Q7TMG8	Nipsnap2	Glioblastoma amplified sequence	281	32.9	7
Q9CYR6	Pgm3	Phosphoacetylglucosamine mutase	542	59.4	6
O88520	Shoc2	Leucine-rich repeat protein SHOC-2	582	64.9	6
P62748	Hpcal1	Hippocalcin-like protein 1	193	22.3	6
Q32ME0	Kcnh6	potassium voltage-gated channel, subfamily H (Eag-related), member 6	950	105.5	6
Q8R5C5	Actr1b	Beta-centractin	376	42.3	6
D3YYS6	Mgll	Monoglyceride lipase	331	36.6	6
D3YWD3	Tmem245	Transmembrane protein 245	880	97.7	6
Q9CPW4	Arpc5	Actin-related protein 2/3 complex subunit 5	151	16.3	6
Q9DAW9	Cnn3	Calponin-3	330	36.4	6
A2A5Y6	Mapt	Microtubule-associated protein	749	78	6
P62827	Ran	GTP-binding nuclear protein Ran	216	24.4	6
Q9CR21	Ndufab1	Acyl carrier protein, mitochondrial	156	17.4	6
Q8R526	Pkd1l1	Polycystic kidney disease protein 1-like 1	2,615	290.7	6
B7ZCF1	Psmc3	26S proteasome regulatory subunit 6A	451	50.4	6
E9Q3L4	Ifi207	Interferon-activated gene 207	978	104.4	6
Q78Y63	Pdcl2	Phosducin-like protein 2	240	27.8	6
E9Q6Y4	Zfp94	Zinc finger protein 94	486	55.5	6
P51163	Uros	Uroporphyrinogen-III synthase	265	28.5	6
P11352	Gpx1	Glutathione peroxidase 1	201	22.3	6
A2A432	Cul4b	Cullin-4B	970	110.6	6
Q923D2	Blvrb	Flavin reductase (NADPH)	206	22.2	6
P51855	Gss	Glutathione synthetase	474	52.2	6
P35505	Fah	Fumarylacetoacetase	419	46.1	6
Q9D892	Itpa	Inosine triphosphate pyrophosphatase	198	21.9	6
Q4VAE3	Tmem65	Transmembrane protein 65	234	24.9	6
H3BLL2	Atpaf1	ATP synthase mitochondrial F1 complex assembly factor 1	348	38.8	6
Q6NZN1	Pprc1	Peroxisome proliferator-activated receptor gamma coactivator-related protein 1	1,644	175	6
Q9JII6	Akr1a1	Alcohol dehydrogenase [NADP (+)]	325	36.6	6
Q9D8S4	Rexo2	Oligoribonuclease, mitochondrial	237	26.7	6
Q8BHE8	Maip1	*m*-AAA protease-interacting protein 1, mitochondrial	291	33	6
Q8VCC2	Ces1	Liver carboxylesterase 1	565	62.6	6
E9PWM3	Armcx4	Armadillo repeat-containing, X-linked 4	2,356	242.8	6
E9QJT5	Acyp1	Acylphosphatase	157	17.3	6
Q9JKL5	Tesc	Calcineurin B homologous protein 3	214	24.6	6
Q2NL51	Gsk3a	Glycogen synthase kinase-3 alpha	490	51.6	6
Q8BXK9	Clic5	Chloride intracellular channel protein 5	251	28.3	6
E9QP56	Apoc3	Apolipoprotein C-III	137	15.2	6
Q9D1X0	Nol3	Nucleolar protein 3	220	24.6	6
Q9WUM4	Coro1c	Coronin-1C	474	53.1	6
Q9JLH8	Tmod4	Tropomodulin-4	345	39.2	6
E9PVA8	Gcn1	eIF-2-alpha kinase activator GCN1	2,671	292.8	6
Q8VE22	Mrps23	28S ribosomal protein S23, mitochondrial	177	20.3	6
Q6P9P0	Slf2	SMC5-SMC6 complex localization factor protein 2	1,278	143.9	6
P04938	Mup11	Major urinary protein 11	181	20.7	6
P26883	Fkbp1a	Peptidyl-prolyl *cis*-trans isomerase FKBP1A	108	11.9	6
P00687	Amy1	Alpha-amylase 1	511	57.6	6
P48542	Kcnj6	G protein-activated inward rectifier potassium channel 2	425	48.6	6
Q9CQ65	Mtap	S-methyl-5′-thioadenosine phosphorylase	283	31	6
S4R1W1	Gm3839	Glyceraldehyde-3-phosphate dehydrogenase	333	35.8	6

**TABLE 2:** Left ventricular tissue proteins ubiquitously identified in both groups but altered with exercise. Only proteins upregulated in ≥6 samples in each group are listed.

UniProt accessions	Description	Gene	Exercised % of control ± SE	q-value
A3KGU9	Spectrin alpha chain, non-erythrocytic 1	Sptan1	24.5 ± 2.4	0.0160
P48771	Cytochrome c oxidase subunit 7A2, mitochondrial	Cox7a2	718.8 ± 49.8	0.0100
P82347	Delta-sarcoglycan	Sgcd	471.4 ± 41.7	0.0133
P17182	Alpha-enolase	Eno1	19.4 ± 1.1	0.0150
Q9Z1J3	Cysteine desulfurase, mitochondrial	Nfs1	231.3 ± 12.7	0.0120
E9Q7L0	Oxoglutarate dehydrogenase-like	Ogdhl	19.2 ± 2.6	0.0120
Q62261	Spectrin beta chain, non-erythrocytic 1	Sptbn1	19.9 ± 2.5	0.0223
Q61234	Alpha-1-syntrophin	Snta1	342.6 ± 26.1	0.0230
Q8BTM8	Filamin-A	Flna	13.8 ± 1.7	0.0213
Q3UIZ8	Myosin light chain kinase 3	Mylk3	6.8 ± 1.1	0.0196
Q8BIJ6	Isoleucine--tRNA ligase, mitochondrial	Iars2	27.0 ± 3.8	0.0193
O70622	Reticulon-2	Rtn2	46.4 ± 5.2	0.0183
H7BX01	Dynamin-like 120 kDa protein, mitochondrial	Opa1	46.1 ± 5.0	0.0175
Q9WUR2	Enoyl-CoA delta isomerase 2, mitochondrial	Eci2	302.7 ± 23.1	0.0171
G3X9J1	Sodium/calcium exchanger 1	Slc8a1	32.9 ± 5.2	0.0168
A0A0R4J0P1	Acyl-coenzyme A dehydrogenase family, member 8	Acad8	43.2 ± 2.9	0.0165
P45952	Medium-chain specific acyl-CoA dehydrogenase, mitochondrial	Acadm	160.0 ± 5.1	0.0170
Q924X2	Carnitine O-palmitoyltransferase 1, muscle isoform	Cpt1b	46.2 ± 3.9	0.0189
P17710	Hexokinase-1	Hk1	33.7 ± 5.5	0.0198
P51881	ADP/ATP translocase 2	Slc25a5	585.8 ± 63.6	0.0200
Q8BH80	Vesicle-associated membrane protein, associated protein B and C	Vapb	367.4 ± 41.3	0.0198
Q9D6J6	NADH dehydrogenase [ubiquinone] flavoprotein 2, mitochondrial	Ndufv2	355.7 ± 39.3	0.0195
P28474	Alcohol dehydrogenase class-3	Adh5	282.7 ± 24.5	0.0191
G3X977	Inter-alpha trypsin inhibitor, heavy chain 2	Itih2	14.8 ± 3.6	0.0200
Q9DCS9	NADH dehydrogenase [ubiquinone] 1 beta subcomplex subunit 10	Ndufb10	372.6 ± 37.2	0.0200
O55143	Sarcoplasmic/endoplasmic reticulum calcium ATPase 2	Atp2a2	61.1 ± 3.9	0.0192
Q3TXS7	26S proteasome non-ATPase regulatory subunit 1	Psmd1	33.7 ± 4.4	0.0197
P29758	Ornithine aminotransferase, mitochondrial	Oat	217.3 ± 16.4	0.0196
P09055	Integrin beta-1	Itgb1	13.2 ± 2.1	0.0207
Q9DCT2	NADH dehydrogenase [ubiquinone] iron-sulfur protein 3, mitochondrial	Ndufs3	596.9 ± 70.5	0.0200
Q02053	Ubiquitin-like modifier-activating enzyme 1	Uba1	14.3 ± 1.8	0.0194
Q9R069	Basal cell adhesion molecule	Bcam	37.8 ± 3.6	0.0190
Q792Z1	MCG140784	Try10	610.5 ± 70.3	0.0188
Q9JKB1	Ubiquitin carboxyl-terminal hydrolase isozyme L3	Uchl3	1,373.5 ± 202.3	0.0187
Q8K2B3	Succinate dehydrogenase [ubiquinone] flavoprotein subunit, mitochondrial	Sdha	49.5 ± 2.3	0.0183
P10493	Nidogen-1	Nid1	46.2 ± 4.5	0.0181
Q8VDM4	26S proteasome non-ATPase regulatory subunit 2	Psmd2	44.4 ± 1.4	0.0182
O35639	Annexin A3	Anxa3	541.7 ± 73.5	0.0178
Q60597	2-oxoglutarate dehydrogenase, mitochondrial	Ogdh	22.6 ± 2.5	0.0200
Q64727	Vinculin	Vcl	47.4 ± 7.3	0.0196
Q6PB66	Leucine-rich PPR motif-containing protein, mitochondrial	Lrpprc	8.5 ± 1.4	0.0198
P28665	Murinoglobulin-1	Mug1	19.5 ± 2.7	0.0196
P62908	40S ribosomal protein S3	Rps3	242.7 ± 16.5	0.0193
Q99LX0	Protein/nucleic acid deglycase DJ-1	Park7	493.9 ± 74.9	0.0197
Q99LC5	Electron transfer flavoprotein subunit alpha, mitochondrial	Etfa	223.7 ± 16.5	0.0196
Q91VM9	Inorganic pyrophosphatase 2, mitochondrial	Ppa2	394.2 ± 43.7	0.0192
Q61941	NAD(P) transhydrogenase, mitochondrial	Nnt	48.3 ± 5.6	0.0188
P70670	Nascent polypeptide-associated complex subunit alpha, muscle-specific form	Naca	51.6 ± 4.2	0.0187
P18242	Cathepsin D	Ctsd	346.1 ± 39.6	0.0196
Q8K1M3	Protein kinase, cAMP dependent regulatory, type II alpha	Prkar2a	39.0 ± 2.5	0.0197
P10605	Cathepsin B	Ctsb	1895.7 ± 357.0	0.0196
P01027	Complement C3	C3	30.8 ± 7.2	0.0192
P61922	4-aminobutyrate aminotransferase, mitochondrial	Abat	22.1 ± 2.0	0.0193
Q9D880	Mitochondrial import inner membrane translocase subunit TIM50	Timm50	28.7 ± 3.0	0.0190
Q922B2	Aspartate--tRNA ligase, cytoplasmic	Dars	23.8 ± 3.4	0.0190
O55234	Proteasome subunit beta type-5	Psmb5	568.5 ± 69.7	0.0191
Q9D8N0	Elongation factor 1-gamma	Eef1g	52.6 ± 3.5	0.0189

Furthermore, all proteins upregulated or identified only in samples of exercised mice were classified into several major classes of proteins (Figures 1D–F). Only those classes represented with at least 3 different proteins are shown in Figure 1D. Exercise increases the metabolic rate in order to meet the energy demand. Thus, as predicted, more than 40% of proteins belong to the metabolite interconversion enzyme class. Also, close to 16% of proteins upregulated with swimming exercise belong to the cytoskeletal protein class (Figure 1D).

To understand the effect of exercise on the transcription of proteins, the identified proteins specified above in the ventricular samples of exercised mice were searched for their known molecular function (Figure 1E) and biological processes (Figure 1F), using the PANTHER classification system. The molecular function of these proteins was mostly involved in catalytic activity (51.0%) and binding (32.7%) (Figure 1E). The cellular processes were primarily classified as various cellular processes (29.3%) and metabolic processes (22.2%). Additionally, more than 10% of proteins have a role in biological regulation and cellular component organization or biogenesis (Figure 1F).

The pathway analysis of the ventricular proteins altered with swimming exercise using Reactome (Figure 1G) suggested that significantly affected pathways were tRNA aminoacylation, pyrophosphate hydrolysis, glutathione conjugation, and respiratory electron transport (Entities FDR <0.03 for all).

Exercise is marked, among others, with elevated ROS production that can lead to peroxidation of lipids, yielding biologically active reactive aldehyde 4-HNE (Al-Menhali et al., 2020). Previously, it was shown that 4-HNE regulates metabolism in the skeletal muscle cells either directly or by adducting to proteins (Al-Menhali et al., 2020). In the present work, modification of proteins by 4-HNE was determined to gain insight into the effects of long-term intensive swimming exercise on the left ventricle and on the potential signaling role of 4-HNE (Table 3). Thirteen different proteins were found to be prone to exercise-induced 4-HNE modifications, compared to eight proteins in the control group. Among the identified 4-HNE–modified proteins, only three proteins were found to be commonly modified for both groups. Those proteins are myoglobin, sarcoplasmic/endoplasmic reticulum calcium ATPase 2 (SERCA2), and laminin subunit alpha-1.

**TABLE 3:** 4-HNE–modified proteins in the left ventricular samples of the control and exercised groups. The table shows the UniProt accession number, main description, gene coding of the protein, number of amino acids (AAs), molecular weight (MW), and number of samples in the control or exercised group having the same protein.

UniProt accession	Description	Gene	# AAs	MW [kDa]	Number of positive samples	
					Control	Exercised
**P04247**	Myoglobin	Mb	154	17.1	6	3
O55143	Sarcoplasmic/endoplasmic reticulum calcium ATPase 2	Atp2a2	1,044	114.8	6	5
P19137	Laminin subunit alpha-1	Lama1	3,084	338	5	5
P58854	Gamma-tubulin complex component 3	Tubgcp3	905	103.4	7	n.d
A7L9Z8	Calcium-transporting ATPase type 2C member 2	Atp2c2	944	102.5	5	n.d
E9QA22	Zinc finger protein 644	Zfp644	1,323	148.3	6	n.d
Q60739	BAG family molecular chaperone regulator 1	Bag1	355	39.7	5	n.d
Q80Y72	Cystatin-like 1	Cstl1	140	16.2	5	n.d
D3YWD3	Transmembrane protein 245	Tmem245	880	97.7	n.d	6
K7N6Y5	Predicted gene 8,011 (fragment)	Gm8011	207	24.8	n.d	5
Q9D8S4	Oligoribonuclease, mitochondrial	Rexo2	237	26.7	n.d	5
E9Q8I0	Complement factor H	Cfh	1,252	141.2	n.d	5
Q3UPL0	Protein transport protein Sec31A	Sec31a	1,230	133.5	n.d	5
P35918	Vascular endothelial growth factor receptor 2	Kdr	1,367	152.4	n.d	5
Q8CFN8	Serine/threonine-protein kinase pim-1	Pim1	313	35.4	n.d	5
Q9WUM4	Coronin-1C	Coro1c	474	53.1	n.d	5
E9Q557	Desmoplakin	Dsp	2,883	332.7	n.d	5
Q6P1G2	Lysine-specific demethylase 2B	Kdm2b	1,309	149.6	n.d	5

## Discussion

In the past decade, significant advances have been made to understand the effects of exercise on the heart. This study aimed to identify alterations in the left ventricle proteome after long-term intensive swimming exercise training. Proteomics depends mainly on mass spectrometry, and by the use of high-resolution instruments and appropriate search engines, changes in the proteome can be detected (Al-Thani et al., 2018). Our earlier study on the erythrocyte proteome demonstrated that besides information on the comparative and quantitative proteome changes, high resolution mass spectrometry is also a vital tool for redox proteomics (Al-Thani et al., 2018). In addition, we have demonstrated that redox modifications of proteins can alter their function (Ludtmann et al., 2018). 4-HNE modification of proteins is one of the common types of oxidative modifications, and the preferred mass spectrometry methods for the detection and identification of proteins modified by 4-HNE use neutral loss-triggered electron capture dissociation tandem mass spectrometry (Rauniyar et al., 2009), collision-induced dissociation (CID), and electron transfer dissociation (ETD) MS/MS (Fritz et al., 2012). We therefore employed a high-resolution tribrid mass spectrometer using HCD and ETD fragmentation methods to investigate changes in the proteome of the left ventricle of the heart, including the identification of 4-HNE–modified proteins, of control and exercised mice. According to the study published several months ago, it is possible that lowering the value for the mass resolution of the mass spectrometer would lead to better protein identification (Sadygov, 2020). In addition, proteins with modifications that were not specified in the database search may result in the lack of protein identification. This study identified 37 proteins upregulated with exercise in addition to 65 proteins identified only in exercised mice samples and also demonstrated a distinct proteome signature of the hearts of exercised and sedentary animals. Indeed, long-term endurance exercise triggers significant physiological and autonomic adaptations to maintain the cardiac function (Rivera-Brown and Frontera, 2012; Gourine and Ackland, 2019). Several studies have demonstrated the effect of endurance exercise–induced transcriptional regulations with regard to increasing mitochondrial biogenesis. The training protocol used in this study increases mitochondrial biogenesis, mitochondrial volume density and number, and both basal and insulin-stimulated glucose uptake in the left ventricle (Vettor et al., 2014). In line with our previous work using this training protocol model (D’Souza et al., 2017; D’Souza et al., 2014; Bidaud et al., 2020), the training efficiency in this study was also evidenced by the development of phenotypic characteristics of the ‘athletes heart,’ including ventricular hypertrophy determined by echocardiography and sinus bradycardia determined by ECG. The cardiac response to swim training observed in this study was similar to that produced by treadmill running in rats where the training status was assessed by measuring the maximal oxygen uptake (VO_2max_) (D'Souza et al., 2017). This study confirmed that more than 10% of proteins identified only after exercise or upregulated proteins in the ventricular tissue of exercised animals have a role in biological regulation and cellular component organization or biogenesis. Moreover, the transcriptional coactivator peroxisome proliferator-activated receptor-γ coactivator-1α (PGC-1α), which is a master regulator of mitochondrial biogenesis, is found to be activated in the hearts of mice after endurance swimming exercise (Boström et al., 2010). In addition, we also found peroxisome proliferator-activated receptor gamma coactivator-related protein one in the exercised group but not in the control group. During exercise, the continuous energy demand is regulated with adequate oxygen supply to support oxidative phosphorylation (Rivera-Brown and Frontera, 2012). This study identified a number of proteins significantly specific to the left ventricle of the exercised group that are directly involved in the mitochondrial electron transport, including acyl carrier protein, ATP synthase mitochondrial F1 complex assembly factor 1, NADH dehydrogenase [ubiquinone] flavoprotein 2, NADH dehydrogenase [ubiquinone] one beta subcomplex subunit 10, NADH dehydrogenase [ubiquinone] iron-sulfur protein 3, cytochrome c oxidase subunit 7A2, cytochrome c oxidase protein 20 homolog, and electron transfer flavoprotein subunit alpha. This is in agreement with earlier studies that reported exercise-induced remodeling of the cardiac mitochondrial proteome (Sun et al., 2008; Kavazis et al., 2009), along with exercise-induced reprogramming of the cardiac mitochondrial phosphoproteome (Ferreira et al., 2014), suggesting that these changes contribute to the cardioprotective phenotype. Mitochondria are one of the main sites of intracellular ROS production and, thus, the main contributors to exercise-induced oxidative stress. Exercise-induced oxidative stress could lead to elevated 4-HNE modulating various cellular processes. Acute 4-HNE exposure can lead to transient adaptation (Al-Menhali et al., 2020), while 4-HNE accumulation, reaching larger concentrations, can have negative effects on cells (Elrayess et al., 2017; Jaganjac et al., 2017). We have found that the glutathione conjugation pathway which represents the main route for 4-HNE detoxification is upregulated with exercise. The glutathione system is among the major endogenous antioxidants (Jaganjac et al., 2020b), and also, the conjugation of the highly reactive carbonyl group in 4-HNE with reduced glutathione is one of the main routes for 4-HNE removal (Esterbauer et al., 1991; Dalleau et al., 2013). Enzymatic reactions can accelerate 4-HNE conjugation *via* enzymes including glutathione-S-transferases (GSTs), alcohol dehydrogenases, and aldehyde dehydrogenases (Chapple et al., 2013). Here, we report that GSTs and alcohol dehydrogenases are upregulated by exercise, suggesting elevated conjugation of 4-HNE. However, the cardiac tissue of the exercised group only had a minor increase in the number of 4-HNE–modified proteins after 5 months of swimming compared to the control group. Studies in animals have shown that long-term exercise reduces protein carbonyls in the plasma and gastrocnemius muscles’ mitochondria (Huertas et al., 2017) and liver mitochondria (Lima et al., 2013), while no differences were found in cardiac tissue (Vieira-Souza et al., 2021). This could be due to cellular adaptation triggered by long-term endurance exercise, which, among others, cases the formation of mitochondrial supercomplexes (Greggio et al., 2017; Huertas et al., 2017), which modulate the generation of mitochondrial ROS, preventing an increase in lipid peroxidation (Barranco-Ruiz et al., 2017). Contrarily, as acute exercise-induced oxidative stress results in elevated oxidation of lipids yielding reactive aldehydes (Tofas et al., 2020), it would probably lead to identification of a larger number of 4-HNE–modified proteins. The proteins myoglobin, SERCA2, and laminin subunit alpha-1, modified by 4-HNE, were detected in the samples of both the control and exercise groups, advocating an important physiological role of 4-HNE. Interestingly, an earlier study on control and diabetic mice hearts, by using Western blot analysis, reported that 4-HNE does not form adducts with SERCA2 (Moore et al., 2013). This discrepancy could be due to the different method sensitivities as the MS/MS ESI IonTrap sensitivity of the Orbitrap Fusion is 100 fg, compared to nanograms for the Western blot used in the earlier study. Moreover, several residues of sarcoplasmic reticulum (SR) vesicles are targets for 4-HNE, and 4-HNE modification of the protein inhibits maximal ATPase activity of SERCA1a. However, the inhibition effect was achieved by using high 4-HNE concentrations (hundreds of μM), which might not reflect the physiological levels of 4-HNE (Hortigón-Vinagre et al., 2011).

Among the proteins modified by 4-HNE in the exercised group were mitochondrial oligoribonuclease and serine/threonine-protein kinase pim-1, both required for the maintenance of the mitochondrial structure and function (Bruni et al., 2013; Din et al., 2013). Indeed, our recent work has demonstrated an important function of 4-HNE on the regulation of mitochondrial metabolism and cellular energy production as well as in inducing adaptation when present at physiological levels (23). In addition, calcium-dependent upregulation of the mitochondrial metabolism involves lipid peroxidation and, in particular, 4-HNE, which might explain 4-HNE modification of calcium-dependent protein binding protein transport protein Sec31A. Exercise-induced 4-HNE modification of desmoplakin and lysine-specific demethylase 2B could suggest 4-HNE involvement in cardiac development (Garcia-Gras et al., 2006). 4-HNE modification of coronin-1C, also observed in this study, was reported to have a role in phagocytosis (Chacko et al., 2016), which is increased by exercise. Vascular endothelial growth factor receptor 2 (VEGFR2) is another protein modified by 4-HNE in the left ventricles of trained mice. Low levels of 4-HNE (0.5–5 μM) upregulate VEGF in retinal endothelial cells and were suggested to promote angiogenesis (Vatsyayan et al., 2012); however, at high levels, 4-HNE (75 μM) was shown to downregulate the expression of VEGFR2, decreasing angiogenesis (Roy and Palaniyandi, 2020). Still, the underlying mechanisms of the role of 4-HNE in the angiogenic response to physical activity *via* the VEGF pathway (Delavar et al., 2014) are yet to be elucidated

## Conclusion

In summary, this study highlights the significant effect of exercise training on mitochondrial biogenesis and the antioxidant defense system. Among the biochemical pathways regulated by proteins upregulated after long-term intensive exercise are respiratory electron transport and glutathione conjugation, while identification of proteins modified by 4-HNE suggest its role in adaptation. These data provide further evidence for the adaptive and beneficial role of physical exercise in maintaining cardiovascular health.

## Data Availability

The datasets presented in this study can be found in online repositories. The names of the repository/repositories and accession number(s) can be found below: https://www.ebi.ac.uk/pride/archive/, PXD026558.
